# Comparison of the Use of H1N1 and seasonal influenza vaccinations between veterans and non-veterans in the United States, 2010

**DOI:** 10.1186/1471-2458-13-1082

**Published:** 2013-11-20

**Authors:** Claudia Der-Martirosian, Kevin C Heslin, Michael N Mitchell, Karen Chu, Kim Tran, Aram Dobalian

**Affiliations:** 1 Veterans Emergency Management Evaluation Center (VEMEC), Department of Veterans Affairs, North Hills, California, USA; 2 School of Nursing, University of California, Los Angeles, USA; 3 Fielding School of Public Health, University of California, Los Angeles, USA

**Keywords:** H1N1, Seasonal flu shot, Veterans, non-Veterans, flu vaccination

## Abstract

**Background:**

Veterans of the U.S. armed forces tend to be older and have more chronic health problems than the general adult population, which may place them at greater risk of complications from influenza. Despite Centers for Disease Control and Prevention (CDC) recommendations, seasonal influenza vaccination rates for the general adult population remain well below the national goal of 80%. Achieving this goal would be facilitated by a clearer understanding of which factors influence vaccination.

**Methods:**

Using the 2010 U.S. National Health Interview Survey (NHIS), this study estimates models of two types of vaccinations (H1N1 and seasonal flu), assesses if the correlates differ for these vaccinations, and analyses the distribution of the correlates by veteran status.

**Results:**

Veterans, women, non-Hispanic whites, non-smokers, those at high risk, educated, with health insurance, and who use clinics as a usual source of care were *more* likely to receive both types of vaccinations. Those who were older, married, and with higher income were *more* likely to get vaccinated for seasonal flu, but not for H1N1. Age and number of children living in the household were found to have different effects for H1N1 compared to seasonal flu.

**Conclusion:**

Veterans are more likely to get vaccinated for seasonal influenza and H1N1 compared to the general population. This might be due to Veterans having better access to care or Veterans participating in better health care practices. Future studies should examine potential differences in flu vaccination use among Veterans using Veterans Affairs (VA) health care system vs. non-VA users.

## Background

Influenza outbreaks occur nearly every year, and cost the U.S. economy an estimated $71-167 billion per year in health services use and lost productivity [1]. Influenza-related diseases also cause an estimated 225,000 hospitalizations and 36,000 deaths annually in the U.S., mostly among chronically ill or elderly people [2-4]. In 2010, the Centers for Disease Control and Prevention (CDC) and the Advisory Committee on Immunization Practices (ACIP) recommended annual influenza vaccination for all persons aged ≥6 months [5], and this recommendation still continues to be in effect [6]. Despite the ACIP recommendations, vaccination rates among the general population remain well below the national Healthy People 2020 goal of 80% [7]. Studies show that vaccination use among high-risk groups in the U.S. remains suboptimal, ranging between 45% and 68% [2,8-12]. Similarly, during the H1N1 epidemic in 2009 H1N1 vaccination rates were below national goals in the U.S., only about 50% of those with diabetes and chronic lung diseases and less than 40% of adults with asthma were vaccinated by mid-November 2009 [5,13-15].

H1N1 and seasonal flu vaccination is critical for all Veterans of the U.S. armed forces, who tend to be older than the general adult population [16], and especially for VA users who are more likely to suffer from multiple health conditions compared to the general population [17]. According to the U.S. Census Bureau, there are approximately 22 million veterans in the U.S., and about a quarter use the VA health care system nationwide [18]. Previous work has shown that the prevalence of influenza vaccination among VA system users is approximately 75% [19,20]. Even though about three quarters of the U.S. Veteran population does not use VA services, previous work on the prevalence of vaccination among Veterans has, to our knowledge, focused almost exclusively on those who use VA services. Further, even though adult immunization use among VA patients is higher than it is in the general adult population, these immunization rates are still not optimal [19,20]. Similarly, in a nationally representative sample of adults with diabetes, Lynch et al. [21] illustrate that even though Veterans with diabetes were more likely to get vaccinated (than were non-Veterans with diabetes) after adjusting for relevant confounding factors, vaccination rates among adult Veterans with diabetes were below the national goal (68.2% for Veterans vs. 31.8% for non-Veterans). Achieving the Healthy People 2020 set an 80% goal for vaccination [7] is difficult without having a clear understanding of the factors that influence vaccination, which may also differ between Veterans and non-Veterans. Previous studies have found that several demographic and behavioral factors are associated with vaccination specifically, age [22], race/ethnicity [10,23], marital status [24], education [24], and smoking [24].

Veterans include a range of persons with a high level of comorbid health conditions (e.g., higher rates of smoking, substance use disorders and other conditions compared to the general adult population) and other socioeconomic factors (e.g., homelessness) that place them among the more vulnerable members of the population [25]. Information on flu vaccination uptake comparing Veterans and non-Veterans is limited, especially on a nationally representative sample. Moreover, if the determinants of H1N1 and seasonal influenza vaccination use are different, then policies and programs aimed at increasing uptake of vaccination will need to differ in terms of outreach, resource allocation, and other characteristics. Using data from the 2010 U.S. National Health Interview Survey (NHIS) and applying the Andersen Behavioral Model of Health Services Use [26,27], this study estimates models of two types of vaccinations (H1N1 and seasonal flu), assesses if the correlates differ for these vaccinations, and analyzes the distribution of the correlates by veteran status.

## Methods

Data from the 2010 NHIS was used for this study. The NHIS is an annual survey conducted by the CDC’s National Center for Health Statistics [28] that provides information on the health status of the U.S. non-institutionalized civilian population through personal household interviews. The NHIS is the nation’s largest household health survey, providing data on health trends, barriers to care, health status, health related behaviors, and risk factors in the U.S. The NHIS is composed of several distinct datasets, of which three were used in this study: the Sample Adult Public Use File, the Persons Public Use File, and the Family Public Use File. The merging of these three data sets yielded a sample of 27,157 unique individuals. This study focuses on 1,342 Veterans who reported having been honorably discharged from active duty in the U.S. armed forces and 13,997 non-Veterans. The analytic sample was limited to non-missing data for the H1N1 question. Missingness was by design since the H1N1 question was removed from the NHIS in August 2010, which coincides with the end of the 2009 H1N1 pandemic globally.

For this study, analyses of these public use data were exempted from review by the Institutional Review Board of the Department of Veterans Affairs Greater Los Angeles Healthcare System.

### Dependent variables

Influenza vaccination status was assessed in the NHIS with the following questions: “During the past 12 months, have you had a seasonal flu shot?” and “Since October 2009, have you had a H1N1 flu vaccination?” The 2010 NHIS was the first year that collected information on H1N1 vaccination rates.

### Independent variables

Age, sex, race/ethnicity, marital status, number of children under age of 18 living in household, education, and current smoking status represent the predisposing domain of the Andersen Model. Enabling factors included insurance coverage, annual household income, employment status, and usual source of care. The need domain of the model is represented by a derived variable on health risks that reflects the CDC’s ACIP recommended groups for vaccination. High-risk individuals were defined as respondents who had any of the following conditions: asthma, cerebral palsy, epilepsy, stroke, intellectual disability, disabling birth defects, lung/breathing problems, emphysema, heart disease, angina pectoris, myocardial infarction, coronary heart disease, blood disorders, metabolic disorders, diabetes, weak/failing kidneys, liver disorders, hepatitis, cancer, arthritis, and current pregnancy.

All of the explanatory variables were categorical. The coding of the explanatory variables is illustrated in Table 1. Age was categorized into four groups: 18–24, 25–44, 45–64 and 65 and over. Mutually exclusive categories were constructed for number of children under age of 18 in household (none, 1–2 children, 3 children or more), education (did not complete high school, high school graduate or GED, some college, and college graduate or beyond), health insurance type (none, private, public/military), family income ($0-$14,999, $15,000-$34,999, $35,000 and more), and usual source of care (clinic, MD office, neither clinic or MD office, none).

**Table 1:** Selected characteristics by Veteran status

**Characteristic**		**Veterans**	**Non-Veterans**	**p-value**^**c**^
**Use of services (Dependent variables):**		**%**^**a**^	**%**^**a**^	
H1N1 Vaccination		24.3	18.7	<0.001
Seasonal Flu Vaccination		49.0	32.7	<0.001
**Predisposing Variables:**				
Age	18–24	7.9	34.1	<0.001
Categories	25–44	29.7	40.0	
	45–64	23.4	12.4	
	65 & up	39.0	13.4	
Gender	Male	92.9	44.0	<0.001
Race	White	80.7	66.5	<0.001
	Black	11.0	12.3	
	Hispanic	6.0	15.4	
	Other	2.3	5.8	
Marital	Married	64.0	53.6	<0.001
Status	Divorced/Separated/Widowed	25.5	18.7	
	Never Married/Single	10.5	27.7	
Children	None	79.6	60.4	<0.001
(< 18) Living	1–2 Children	17.2	30.5	
in Household	3 or More Children	3.2	9.1	
Education	Did not complete high school	8.3	15.2	<0.001
	High school graduate or GED	30.3	26.7	
	Some college	35.6	30.5	
	College graduate or beyond	25.9	27.6	
Smoking	Yes	22.4	19.7	0.041
**Enabling Variables:**				
Health	None	8.6	19.8	<0.001
Insurance	Private	35.9	55.5	
	Public/Military	55.5	24.8	
Family	$0–$14,999	27.5	33.7	<0.001
Income	$15,000–$34,999	17.5	14.7	
	$35,000 & up	55.0	51.6	
Employed	Yes	45.7	61.9	<0.001
Usual	Clinic	17.7	16.3	<0.001
Source of	MD Office	65.1	62.7	
Care	Neither Clinic/MD Office	8.0	3.5	
	None	9.3	17.5	
**Need Variable:**				
High Risk^b^	Yes	66.0	46.5	<0.001
	Unweighted Sample Size (N = 15339)	1,342	13,997	

^a^Weighted predicted percents of the population.

^b^Currently pregnant, asthma, cerebral palsy, epilepsy, stroke, intellectual disability (mental retardation), birth defect that causes limitations/difficulty.

with activities, lung/breathing problems, emphysema, heart disease, angina pectoris, myocardial infarction, coronary heart disease, blood disorders,

metabolic disorders, diabetes, weak/failing kidneys.

^c^This is the p value of the test of the association of the predictor by veteran status. Normally this would be a chi-square test, but accounting for the survey design (via the “svy” prefix) yields an F-test. The denominator degrees of freedom is 300 (reflecting the sample design) and the numerator degrees of freedom is the number of categories of the predictor minus 1.

### Statistical analysis

All analyses were conducted with the Stata/SE software program (version 12.1). The “svyset” command was used to account for the complex sampling design (i.e., stratification, clustering, and oversampling) according to NHIS documentation [29]. The “svy” prefix was used in conjunction with estimation commands to account for the complex sampling design. Bivariate analyses were conducted to compare the distribution of socio-demographic characteristics and other relevant study variables between the Veteran and non-Veteran populations. Separate multiple logistic regression analyses were conducted with H1N1 flu vaccine (Model 1) and seasonal flu vaccine (Model 2), the two dependent variables. Adjusted odds ratios (ORs) and 95% confidence intervals (CIs) were calculated for each explanatory variable. The two models were then jointly estimated (using the Stata “suest” command) comparing the size of the coefficients associated with H1N1 versus seasonal flu. The level of significance for all analyses was set at 0.05.

## Results

Table 1 compares the characteristics of Veterans and non-Veterans in the U.S. in 2010. The final sample consisted of 1,342 adult Veterans and 13,997 adult non-Veterans. H1N1 and seasonal influenza vaccination prevalence was higher for Veterans compared to non-Veterans (H1N1: 24.3% vs. 18.7%; seasonal flu: 49.0%, vs. 32.7%). Compared to non-Veterans, Veterans were *more* likely to be male, white, older, married, not to have children, unemployed, high school graduate or with some college, have public/military insurance, have any type of usual source of care, and be at high risk for influenza.

### H1N1 Vaccination (model 1)

Table 2 shows the ORs and 95%CIs associated with each explanatory variable from the multivariable logistic regression model predicting H1N1 vaccination (Model 1). The results show that the odds of a Veteran obtaining the H1N1 vaccine was 1.30 times (95% CI (1.09, 1.55), p < 0.01) the odds of a non-Veteran receiving the H1N1 vaccine. Males (OR = 0.73, 95% CI (0.65, 0.82), p < 0.001), blacks (OR = 0.73, 95% CI (0.62, 0.85), p < 0.001), smokers (OR = 0.74, 95% CI (0.64, 0.86), p < 0.001), and those who lacked usual source of care (OR = 0.52, 95% CI (0.43, 0.64), p < 0.001) had *lower* odds of obtaining the H1N1 vaccine. Veterans, college graduates (OR = 1.80, 95% CI (1.49, 2.19), p < 0.001) or some college (OR = 1.29, 95% CI (1.07, 1.55), p < 0.01), private (OR = 1.52, 95% CI (1.25, 1.85), p < 0.001), or public/military health insurance (OR = 1.78, 95% CI (1.44, 2.21), p < 0.001), and those at high risk (OR = 1.51, 95% CI (1.36, 1.67), p < 0.001) had *greater* odds of obtaining the H1N1 vaccine.

**Table 2:** Odds ratios (OR) with 95% confidence intervals (95% CI) from models predicting receipt of H1N1 vaccine (H1N1) and receipt of seasonal flu vaccine (Seasonal)

		**H1N1**		**Seasonal**	
		**OR**	**95% CI**	**OR**	**95% CI**
Veteran	No	Ref		Ref	
Status	Yes	1.30^**^	1.09,1.55	1.42^***^	1.23,1.66
Age	18–24	Ref		Ref	
Categories	25–44	1.04	0.91,1.20	1.19^**^	1.06,1.34
	45–64	1.34^**^	1.10,1.63	1.88^***^	1.63,2.18
	65 up	1.27^*^	1.01,1.59	3.05^***^	2.54,3.65
Gender	Female	Ref		Ref	
	Male	0.73^***^	0.65,0.82	0.65^***^	0.59,0.72
Race	White	Ref		Ref	
	Black	0.73^***^	0.62,0.85	0.81^**^	0.71,0.94
	Hispanic	1.06	0.92,1.22	0.94	0.82,1.07
	Other	1.24^*^	1.03,1.48	1.15	0.98,1.35
Marital	Married	Ref		Ref	
Status	Div/Sep/Wid	0.89	0.77,1.02	0.85^**^	0.75,0.95
	Never/Single	0.93	0.78,1.09	0.78^***^	0.68,0.89
Children	None	Ref		Ref	
< 18 Living	1–2 kids	1.18^*^	1.01,1.37	0.87^*^	0.77,0.97
in HH	3 or more kids	1.15	0.94,1.40	0.84	0.69,1.01
Education	Not HS	Ref		Ref	
	HS Grad	1.06	0.89,1.28	1.04	0.90,1.20
	Some Coll	1.29^**^	1.07,1.55	1.17	0.99,1.37
	Coll Grad	1.80^***^	1.49,2.19	1.58^***^	1.34,1.86
Smoking	No	Ref		Ref	
	Yes	0.74^***^	0.64,0.86	0.69^***^	0.60,0.78
Health	None	Ref		Ref	
Insurance	Private	1.52^***^	1.25,1.85	1.62^***^	1.36,1.93
	Pub/Mil	1.78^***^	1.44,2.21	2.09^***^	1.72,2.55
Family	0–$15 k	Ref		Ref	
Income	$15 k-$35 k	1.02	0.87,1.19	1.14	0.99,1.30
	$35 k+	1.04	0.90,1.20	1.24^***^	1.11,1.40
Employed	No	Ref		Ref	
	Yes	1.00	0.88,1.12	1.07	0.96,1.20
Usual Source	Clinic	Ref		Ref	
of Care	MD Office	0.86^*^	0.76,0.97	1.04	0.91,1.17
	Neither	0.79	0.58,1.08	0.86	0.66,1.11
	None	0.52^***^	0.43,0.64	0.52^***^	0.43,0.63
High Risk	No	Ref		Ref	
	Yes	1.51^***^	1.36,1.67	1.47^***^	1.33,1.63

N = 15339.

^*^p < 0.05, ^**^p < 0.01, ^***^p < 0.001.

### Seasonal Flu vaccination (model 2)

Table 2 shows that Veterans were significantly more likely to obtain the seasonal flu vaccine than non-Veterans. The odds of a Veteran obtaining the seasonal flu vaccine was 1.42 times the odds of a non-Veteran receiving the vaccine (95% CI (1.23, 1.66), p < 0.001). Each age group tested (25–44, 45–64, ≥65) was significantly more likely to get a seasonal flu vaccine compared to the reference group (18–24) (p’s < 0.01). Males (OR = 0.65, 95% CI (0.59, 0.72), p < 0.001), blacks (OR = 0.81, 95% CI (0.71, 0.94), p < 0.01), divorced/separated/widowed (OR = 0.85, 95% CI (0.75, 0.95), p < 0.01), never married/single (OR = 0.78, 95% CI (0.68, 0.89), p < 0.001), smokers (OR = 0.69, 95% CI (0.60, 0.78), p < 0.001), and those with no usual source of care (OR = 0.52, 95% CI (0.43, 0.64), p < 0.001) had *lower* odds of receiving the seasonal flu. College graduates (OR = 1.58, 95% CI (1.34, 1.86), p < 0.001), those with private insurance (OR = 1.62, 95% CI (1.36, 1.93), p < 0.001) or public/military insurance (OR = 2.09, 95% CI (1.72, 2.55), p < 0.001), the highest income group (OR = 1.24, 95% CI (1.11, .40), p < 0.001), and those at high risk (OR = 1.47, 95% CI (1.33, 1.63), p < 0.001) had *greater* odds of receiving the vaccine.

### H1N1 vs. Seasonal Flu vaccination

Table 3 shows tests of the equality of the coefficients from Model 1 (H1N1 flu) and Model 2 (seasonal flu). The effect of age and number of children significantly differed for Model 1 compared to Model 2 (p < 0.01). The overall test comparing the coefficients associated with the four levels of age showed significant differences between Model 1 and Model 2 (p < 0.001). Tests of each of the individual coefficients for age showed that the coefficient comparing ages 25 to 44 to the reference group (18 to 24) was not significant (p = 0.067), however the coefficient for ages 45 to 64 (p < 0.003) and for age 65 and up (p < 0.001) were significant. The effect of being in these older age categories (45–64 and 65&up) as compared to the reference group (18 to 24) was greater for the seasonal flu vaccine compared to the H1N1 vaccine.

**Table 3:** Tests of the equality of the effects (coefficients) from Model 1 (H1N1 flu) compared to Model 2 (seasonal flu)

**Variable**	**df**	**F test**	**p-value**
Veteran Status	1	1.36	.245
Age	3	25.10	<0.001
Gender	1	3.73	.054
Race	3	1.91	.127
Marital Status	2	1.09	.338
Children < 18 in HH	2	9.43	<0.001
Education	3	.88	.452
Family Income	2	2.45	.088
Employed	1	0.73	.394
Health Insurance	1	1.22	.269
Smoking	1	1.35	.247
Usual Source of Care	3	3.71	.012
High Risk	1	0.19	.667

## Discussion

According to predisposing, enabling and need factors identified in the Anderson Behavioral Model, there were significant differences between Veterans and non-Veterans on characteristics such as gender, race, age, marital status, employment status, education, health insurance, type of usual source of care, and high-risk status. These initial findings guided the inclusion of selected set of independent variables in the models analyzing flu vaccination by Veteran status. The results of this study confirm previous findings and provide important additions to the current literature about comparisons in influenza and H1N1 vaccinations among Veterans and non-Veterans using the 2010 NHIS. The findings indicate that Veterans, women, non-Hispanic whites, those with higher education, non-smokers, those with health insurance (private or public/military), those with a clinic as a usual source of care, and those who are high risk were *more* likely to receive both types of vaccinations, which is largely consistent with findings from previous studies [8,10,21,23,24,30]. Age, marital status, and family income were additional significant correlates of using the seasonal flu vaccine, which is also consistent with previous studies [8,24] (i.e. older age groups, those who are married, and those with more income were *more* likely to get vaccinated for seasonal flu). The finding on age may in part reflect the impact of public information campaigns of the Center for Medicare and Medicaid Services, which actively promotes the use of vaccines for seasonal flu [31]. Use of the H1N1 vaccine was not sensitive to differences in income, perhaps because of the urgent nature of the pandemic, i.e. the strength of the perceived threat surpassed concerns about financial cost. Similarly, the social support and encouragement for vaccine use that one receives from a spouse may not be as important when the perceived health threat is relatively high, as was likely the case with the unexpected global pandemic of H1N1.

The dataset used in this study does not include a measure of where the Veteran received a vaccination. The VA is not a health insurance system, but rather a separate integrated healthcare system that is accessible by qualified Veterans. In general, the use of preventive care among VA-users is higher than the general public. This may account for at least some of the relatively higher use of vaccinations among Veterans compared to non-Veterans. Although this finding is encouraging, the use of vaccinations among Veterans is still significantly lower than is recommended by the CDC and more efforts should be made to promote greater use of vaccinations among Veterans.

The study tested the equality of the coefficients for H1N1 and seasonal influenza vaccination. Statistically significant differences in the effect of age and number of children living in the household were found for H1N1 compared to seasonal flu. For seasonal flu, all three older age groups were more likely to receive vaccination compared to 18–24 year olds. For H1N1, however, this pattern was not found – i.e. the older groups were just as likely to get vaccinated as the youngest age group (18–24). It is possible that seasonal flu is generally perceived as a pathogen to which one becomes more susceptible with age, whereas H1N1 is perceived as a threat to all, regardless of age. Similarly, different patterns were found for number of children living in households – those with children were *less* likely to receive seasonal flu vaccination whereas for H1N1, those with children were *more* likely to receive H1N1 vaccination. People with more children may be more so accustomed to dealing with upper respiratory tract infections that seasonal flu might seem more “normal” and manageable, whereas H1N1 might seem more unpredictable and threatening, thus motivating the use of vaccine. Moreover, the aforementioned news reports in 2010 suggesting that H1N1 placed children at greater risk of morbidity or mortality than did seasonal influenza likely motivated parents/guardians to have their children vaccinated for H1N1 [32,33].

There are strengths and limitations of this study. The strengths of this study are that it used a nationally representative sample of both Veterans and non-Veterans providing comparable data on influenza and H1N1 vaccination for both groups. The statistical method used in the analyses reliably mirrored receipt of vaccination for these two groups assessing whether the various indictors of immunization varied between Veterans and non-Veterans. The study has some limitations. Due to lack of data, patient preferences or attitudes towards vaccination were not included in the analyses. Previous research has shown factors such as fear of side effects from vaccination [34], efficacy concerns [34,35], doctor recommendation [34], and the fear that vaccination actually causes influenza [34,36] to have an effect on the likelihood of getting vaccinated. To the extent that any omitted variables are associated with the variables that we were able to include in our analyses, the multivariate models may at least partially account for their effects. Nevertheless, future work should include variables on preferences, attitudes, and other such characteristics whenever possible. Also, potential differences in vaccination use among Veterans using the VA health care system, Veterans not using the VA health care system, and Veterans who use both VA and non-VA health care is certainly worthy of study. Unfortunately, the lack of NHIS data on VA system use does not permit comparisons of vaccination use among these three groups. The data used in the analyses were cross-sectional, limiting the extent to which causality could be assessed; however, reverse causality is not a major concern for the key question about the relationship between Veteran status and vaccination use.

## Conclusion

In summary, the findings from this study show that Veterans are more likely to get vaccinated than are non-Veterans. This suggests that there may be effective ways to improve vaccination rates among other populations that are socioeconomically disadvantaged or otherwise vulnerable. The observed differences in vaccination rates may be due to some Veterans having better access to care because they qualify to use the VA, or other potential differences related to Veterans health care practices and attitudes toward prevention. Future studies should examine whether better access to care for other vulnerable populations can also lead to higher vaccination rates among other groups. Regardless of Veteran status, the determinants of vaccination use are different for H1N1 and seasonal influenza for certain population subgroups, suggesting areas for improving immunization rates among both Veteran and non-Veteran populations. From a policy perspective, the findings on family income and marital status suggest that outreach programs for people from lower-income backgrounds or who may be more isolated (widowed, never married, etc.) would be more effective at improving access to vaccinations for seasonal flu than H1N1. In an integrated delivery system such as the VA, electronic reminder systems have been successfully used at point of care to increase adherence to clinical practice guidelines for a number of health conditions and services, including vaccinations. However, efforts are needed to improve the delivery of care to all Veterans, both VA health care system users and non-users alike.

## Abbreviations

VA: Department of Veterans Affairs; NHIS: National Health Interview Survey; CDC: Centers for Disease Control and Prevention; ACIP: Advisory Committee on Immunization Practices.

## Competing interests

The authors declare that they have no competing interests.

## Authors’ contribution

CDM conceived the study, participated in its design, and drafted the manuscript. KCH participated in its design, assisted in writing and editing the manuscript. MNM performed the statistical analysis and interpreted the findings. KC performed the statistical analysis and edited the manuscript. KT assisted in critically revising the manuscript. AD participated in the design of the study, drafted the manuscript and revised it critically. All authors read and approved the final manuscript.

## Authors’ information

Claudia Der-Martirosian, PhD is a research health scientist at the Department of Veterans Affairs’ (VA) Veterans Emergency Management Evaluation Center (VEMEC) with over 15 years of research experience quantitative research methods. Prior to joining VEMEC, she was a senior statistician at UCLA, UC San Diego, and UC Davis. Her research experience includes one book publication and over 50 first-authored and co-authored publications in health related fields such as public health, quality of life, and medical education.

Kevin C. Heslin, PhD is a research health scientist at VEMEC. His research focuses on access to health services and health outcomes in marginalized populations, particularly among individuals with substance use disorders. With his colleagues in VEMEC, he has conducted work on disaster preparedness among U.S. veterans in California, as well as the differential impact of the 1994 Northridge earthquake on veterans with screening positive for substance abuse or dependence. Currently he is examining the impact of Hurricane Ike on veterans’ access to methadone maintenance therapy in the Houston, Texas area. Dr. Heslin received his Ph.D. from the Department of Health Management and Policy at UCLA, and was a predoctoral fellow in the National Institute of Mental Health/UCLA AIDS Research Training Program.

Michael N. Mitchell, PhD was a senior statistician at VEMEC, at the time this manuscript was developed. He is the author of *Interpreting and Visualizing Regression Models Using Stata*, A *Visual Guide to Stata Graphics*, as well as *Data Management Using Stata*. Previously, he worked for 12 years as a statistical consultant and manager of the UCLA ATS Statistical Consulting Group where he envisioned the UCLA Statistical Consulting Resources website and wrote hundreds of web pages about the use of SAS, Stata, SPSS, and other statistical computing packages.

Karen Chu, MS is a statistician for VEMEC and the VA Health Services Research and Development Center of Innovation (HSR&D). Ms. Chu has a Master of Science in Pharmaceutical Economics and Policy from the University of Southern California. She has experience working with medical claims databases, national survey data, quality of life data, and clinical trial data. She is primarily responsible for coordinating the use of various VA and non-VA datasets for research, extraction, and analysis.

Kim Tran PhD, RN, a nurse practitioner, completed her baccalaureate training in nursing at the University of Southern California in 1997. She completed her doctoral training at the UCLA School of Nursing in 2006 where she also obtained her Master’s degree. Her research interest is in health disparities and examining access to care among vulnerable populations using quantitative and qualitative methods. She has worked in the critical care and coronary care settings as a registered nurse and as a nurse practitioner for over 15 years. Previously, she was an Assistant Professor at California State University of Los Angeles where she taught both undergraduate and graduate nursing students. She was a postdoctoral fellow at the Department of Veterans Affairs HSR &D Center of Excellence for the Study of Healthcare Provider Behavior as well as a UCLA faculty in the School of Nursing at the time this manuscript was developed.

Aram Dobalian, PhD, JD is the founding Director of the U.S. Department of Veterans Affairs’ (VA) Veterans Emergency Management Evaluation Center (VEMEC). VEMEC’s work focuses on cutting-edge policy and operations questions that support VA’s emergency management responsibilities – to improve the Nation’s preparedness for response to war, terrorism, national emergencies, and natural disasters by ensuring continuity of veteran care, and to support national, state, and local emergency management, public health, safety, and homeland security efforts.

## Pre-publication history

The pre-publication history for this paper can be accessed here:

http://www.biomedcentral.com/1471-2458/13/1082/prepub
